# Organ dysfunction, thrombotic events and malignancies in patients with idiopathic multicentric castleman disease: a population-level US health claims analysis

**DOI:** 10.1038/s41375-022-01690-2

**Published:** 2022-09-29

**Authors:** Sudipto Mukherjee, Karan Kanhai, David Kauffman, Rabecka Martin, Jeremy S. Paige, Anirvan Ghosh, Hannah Kannan, Francis Shupo, David C. Fajgenbaum

**Affiliations:** 1 Department of Hematology and Medical Oncology, Taussig Cancer Institute, Cleveland Clinic, Cleveland, OH USA; 2 EUSA Pharma, Hemel Hempstead, UK; 3 Eversana, LLC, Milwaukee, WI USA; 4 EUSA Pharma, Burlington, MA USA; 5 Department of Medicine, Perelman School of Medicine, University of Pennsylvania, Philadelphia, PA USA

**Keywords:** Haematological diseases, Lymphoproliferative disorders

## To the Editor:

Idiopathic multicentric Castleman disease (iMCD) is a rare lymphoproliferative disorder characterized by a hyperinflammatory state driven primarily by hypercytokinemia, particularly interleukin-6 (IL-6) [1–3]. iMCD patients have historically poor outcomes [4], particularly among patients who were not properly treated [5]. Among several factors, one potential reason for delayed treatment initiation could be a lack of understanding of the clinical trajectory of iMCD patients regarding the development of disease-associated morbidities over time. A better understanding of the natural history of iMCD, particularly the evolution and prevalence of morbidities, will be helpful for making timely decisions about appropriate treatment and potentially mitigate morbidity burden in these patients. The primary objective of this study was to evaluate the pattern of morbidities and their prevalence in iMCD patients using a nationally representative health claims dataset. We additionally analyzed healthcare utilization of iMCD patients by evaluating length of hospital stays and visits to emergency departments by these patients.

Anonymized longitudinal patient data were sourced from Truven MarketScan® Research Databases (IBM), covering 235 million US citizens (commercially-insured, on Medicare, or Medicaid) from January 2006 to December 2020 [6]. All eligible iMCD patients were identified using a previously validated claims- based algorithm that in addition to CD-specific ICD-10 diagnosis code (D47.Z2), required negative HHV-8 and HIV status, and presence of corresponding diagnostic or laboratory claims for ≥2 minor criteria, consistent with published diagnostic criteria recommendations for iMCD [7, 8]. A patient’s index diagnosis date (IDD) was defined as the first date of the D47.Z2 diagnosis claims code when available or the previous nonspecific ICD-9-CM code for enlargement of lymph nodes previously used for CD (785.6), whichever appeared first. Morbidities occurring in iMCD patients were grouped in three categories and identified using ICD9/10 diagnosis codes: (1) Organ dysfunction and thrombotic events including heart failure, liver dysfunction, renal dysfunction, respiratory dysfunction or interstitial lung disease, drug-induced diabetes mellitus, and thrombotic events (stroke, transient ischemic attack, extremity deep vein thrombosis, pulmonary embolism, and portal vein thrombosis); (2) Myeloid malignancies including acute myeloid leukemia, chronic myeloid leukemia, myelodysplastic syndromes (MDS), and Philadelphia chromosome-negative myeloproliferative neoplasm (MPN); (3) Solid malignancies including colon, lung, skin (melanoma), breast, prostate, thyroid, head and neck, and carcinoma of unknown primary.

To better understand important features in the longitudinal histories of these patients, we performed a mutual information (MI) analysis of the iMCD population relative to controls. This allowed us to quantify binary features that are statistically most relevant for discriminating between iMCD and non-iMCD patients. The top 15 diagnosis codes by mean MI value across all 6-month periods are provided in Table 1A.

**Table 1:** Common morbidities and healthcare burden in iMCD patients. **A**. Proportion of patients with morbidities in iMCD and non-iMCD cohort identified in Mutual Information analysis. **B**. ER visits and length of inpatient stay for iMCD patients by year relative to index date of diagnosis.

ICD-10 Code	Morbidity	Mean MI Value	iMCD patients	Control patients	p^*^
R59.0	Localized enlarged lymph nodes	0.023	47.2%	0.8%	*<0.001*
R59.9	Enlarged lymph nodes, unspecified	0.020	48.0%	0.3%	*<0.001*
R59.1	Generalized enlarged lymph nodes	0.019	35.8%	0.3%	*<0.001*
D47.Z9	Other specified neoplasms of uncertain behavior of lymphoid, hematopoietic and related tissue	0.011	24.7%	0.0%	*<0.001*
I10	Essential (primary) hypertension	0.009	49.4%	14.8%	*<0.001*
D64.9	Anemia, unspecified	0.009	38.4%	3.5%	*<0.001*
Z79.899	Other long term (current) drug therapy	0.008	43.5%	7.9%	*<0.001*
D50.9	Iron deficiency anemia, unspecified	0.007	23.6%	1.6%	*<0.001*
R91.8	Other nonspecific abnormal finding of lung field	0.007	31.7%	2.2%	*<0.001*
K21.9	Gastro-esophageal reflux disease without esophagitis	0.006	33.9%	7.0%	*<0.001*
R10.9	Unspecified abdominal pain	0.006	33.2%	5.5%	*<0.001*
R50.9	Fever, unspecified	0.005	26.6%	3.2%	*<0.001*
R06.02	Shortness of breath	0.005	32.1%	5.0%	*<0.001*
R07.9	Chest pain, unspecified	0.005	35.8%	5.8%	*<0.001*
G47.33	Obstructive sleep apnea (adult) (pediatric)	0.004	18.5%	3.1%	*<0.001*

	Days admitted to ER/Days of Inpatient stays (*n*)	ER visits *n* (%)	Inpatient length of stay in days *n* (%)
2 years before	0	154 (79.8)	174 (90.2)
	1	17 (8.8)	1 (0.5)
	2	14 (7.2)	1 (0.5)
	3	0 (0)	1 (0.5)
	4	2 (1)	4 (2.1)
	5+	6 (3.1%)	12 (6.2)
1 year before	0	123 (52.1)	117 (49.6)
	1	47 (19.9)	1 (0.4)
	2	31 (13.1)	9 (3.8)
	3	13 (5.5)	15 (6.4)
	4	5 (2.1)	13 (5.5)
	5+	17 (7.2)	81 (34.3)
Year 1	0	136 (57.4)	168 (70.9)
	1	44 (18.6)	2 (0.8)
	2	23 (9.7)	8 (3.4)
	3	10 (4.2)	16 (6.8)
	4	6 (2.5)	6 (2.5)
	5+	18 (7.6)	37 (15.6)
Year 2	0	134 (86.5)	143 (92.3)
	1	15 (9.7)	1 (0.7)
	2	3 (1.9)	1 (0.7)
	3	1 (0.7)	1 (0.7)
	4	1 (0.7)	1 (0.7)
	5+	1 (0.7)	8 (5.2)

Percentage of patients calculated at 24 months.

*ICD* International Classification of Diseases, *MI* mutual information, *ER* emergency room visit, *n* number of patients, *%* percentage.

*p values calculated from Chi-squared test and *p* < 0.001 considered significant.

To analyze disease burden over time, we calculated cumulative proportions of individual morbidities across the group over a 4-year period (±2 years from IDD). To control for confounding, we matched all iMCD patients to non-iMCD controls at a frequency of 1:50 on age group (0–17, 18–44, 45–54, 55–64, >65 years), sex, insurance type, length of claims history in database, and region (Northeast, South, Midwest, West).

The number of ER visits and inpatient hospitalization days per patient, defined by specific place of service codes, were calculated for each patient in 12 months prior to (1 year before) and 12 months following (1 year after) IDD. When available, ER visits and hospitalizations were also calculated for 13–24 months prior to or following (±2 years) IDD. Multiple ER claims on a single day were counted as a single visit. All significance tests for continuous variables were calculated using Welch’s Unequal Variance *T*-Test with a significance level of 0.05. A chi-square test without Yates correction was used to identify significance between features described by binary variables.

Of 30.7 million eligible individuals in the Truven MarketScan® database, 487 patients had a Castleman disease (CD) diagnosis—of which 271 were identified as iMCD based on our algorithm [7]. Compared to the base population (non-CD enrollees), the iMCD cohort had a higher proportion of females (59.4%), higher proportion of private insurance coverage (66.4%) and lower proportion of Medicaid (24.7%), with an average continuous enrolment of 6.7 years (data not shown). Approximately, 90% of iMCD patients in this dataset did not have claims corresponding with IL-6 directed therapies.

We performed an unbiased MI analysis to identify the most common morbidities between iMCD and a non-iMCD control population (Table 1A). The top three morbidities with the highest mean MI value in iMCD patients were localized enlarged lymph nodes (mean MI value 0.023), enlarged lymph nodes (mean MI value 0.020), and generalized enlarged lymph nodes (mean MI value 0.019). These findings are consistent with what we anticipated and support the clinical validity of additional morbidities that were found. Three additional important findings of clinical interest in iMCD patients over 2 years on either side of diagnosis were hypertension, anemia and neoplasms.

Our analysis of iMCD burden over time as a function of ER visits and hospitalization days revealed increased healthcare utilization in the year immediately before and after IDD compared to 2 years before and 2 years after diagnosis (Table 1B). A higher proportion of iMCD patients required an ER visit (47.8% and 42.6% in the year before and after IDD, respectively) before dropping to 13.5% in the second year after diagnosis. The proportion of patients with greater than five ER visits increased from 3.1% 2 years prior to diagnosis to 7.2% and 7.6% in the 2 years around IDD. The same pattern was observed with inpatient stays with the most marked increase observed in those who were hospitalized for at least 5 days, increasing from 6.2% 2 years prior to diagnosis to 34.3% and 15.6% in the subsequent 2 years.

**Fig. 1:**
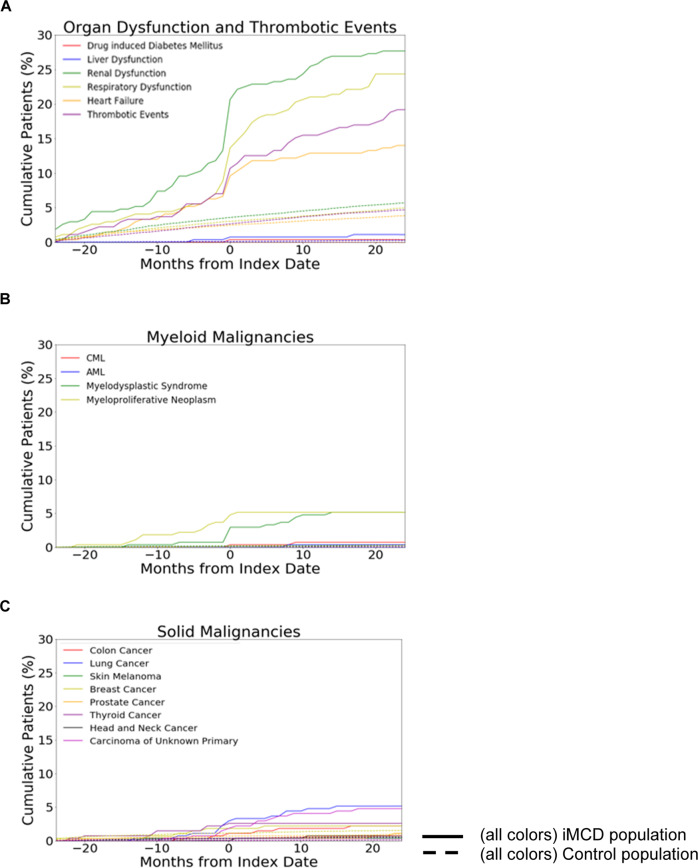
Development of morbidities over time in iMCD patients before and after diagnosis relative to non-disease controls. **A** Cumulative proportion of various organ dysfunction and thrombotic events over time between iMCD patients and non-disease controls, **B** Cumulative proportion of myeloid malignancies over time between iMCD patients and non-disease controls, **C** Cumulative proportion of solid malignancies over time between iMCD patients and non-disease controls.

The cumulative proportion of myeloid malignancies, organ dysfunction, thrombotic events, and solid malignancies were significantly higher in iMCD patients compared to the control patients (*p* < 0.001) (Fig. 1). Among all organ dysfunction-related morbidities, there was a substantially higher proportion of iMCD patients with renal, respiratory dysfunction, and thrombotic events compared to controls (*p* < 0.001) (Fig. 1A). Overall, the cumulative proportion of myeloid and solid malignancies were higher in iMCD patients compared to controls (10.0% vs 0.6%,18.1% vs 7.4%, respectively; *p* < 0.001). Among all myeloid malignancies, MDS and MPN accounted for the highest proportion of cases, whereas carcinoma of unknown primary accounted for the most cases of solid malignancies in iMCD patients (Fig. 1B, C). We additionally undertook analysis of lymphoid malignancies, which revealed higher cumulative proportions of non-Hodgkin’s lymphoma in iMCD patients following IDD compared to control (19.2% vs 0.4% at 24 months post-index) (data not shown). Of note, we observed similar patterns of increasing proportions of morbidities with age across all morbidity categories in both the non-disease and iMCD cohorts.

To the best of our knowledge, this is the first population level study to comprehensively characterize the pattern and evolution of morbidities in iMCD patients leading up to and following iMCD diagnosis. The key findings of this study include a high proportion of iMCD patients experiencing renal and pulmonary dysfunction, thrombotic events, myeloid malignancies particularly MDS and MPN, and solid tumors particularly carcinoma of unknown primary. We observed an increase in morbidities leading up to the iMCD diagnosis and continued rise (either persistence or emergence of new morbidities) post diagnosis even in the short follow-up period (2 years). This correlated with increased healthcare utilization, potentially reflecting difficulties in establishing the diagnosis and contributing to increasing severity at the time of treatment initiation.

Earlier studies have reported an increased prevalence of certain morbidities including a three-fold increased prevalence of malignancy (19%) compared to age-matched controls (6%) in iMCD patients [4, 5, 8, 9]. Our larger comparative analyses using a matched cohort corroborate those earlier observations. However, it is unclear how to interpret the increased lymphoid malignancies as they are iMCD mimics and may represent misdiagnoses or miscoding.

A major strength of this study was reliance on a validated claims-based methodology to identify iMCD cases from a large population cohort and comparison with matched non-iMCD controls, overcoming the limitations of small sample sizes and lack of appropriate control groups seen in previous studies. Given the rarity of this condition, introduction of evidence-based diagnostic criteria and specific disease coding in 2017, and slow recruitment of patients in disease-specific registries, administrative claims databases remain valuable tools for studying natural history and real-world treatment outcomes of rare diseases.

Our study has several limitations inherent to health claims datasets, therefore, the findings should be interpreted with appropriate caution [7]. As claims analyses do not require histopathology confirmation or clinical documentation, iMCD mimics or CD subtypes other than iMCD may have been inadvertently included in this study. Claims data do not have survival information. Apart from information about occurrence of a health event, it does not provide information on subsequent outcomes. Lastly, the MarketScan database provides an over-weighted sample of privately insured individuals and may not be generalizable to uninsured patients or to those who primarily receive care through government insurance programs.

This study enhances our understanding of the natural history of iMCD particularly related to the development of morbidities with implications for early diagnosis and timely treatment. Further research is ongoing using orthogonal approaches such as the ACCELERATE Registry (www.CDCN.org/ACCELERATE) [10] to assess morbidities in iMCD.

## Data Availability

The datasets analyzed during the current study were made available to the authors through licensing from the data provider. The data cannot be made publicly available due to the data use agreement.

## References

[1] Dispenzieri A, Fajgenbaum DC (2020). Overview of Castleman disease. Blood.

[2] Yu L, Tu M, Cortes J, Xu-Monette Z, Miranda R, Zhang J, et al. Clinical and pathological characteristics of HIV- and HHV-8-negative Castleman disease. 2021. https://reference.medscape.com/medline/abstract/28100459 (accessed December 9, 2021). 10.1182/blood-2016-11-748855 PMC5364343 28100459

[3] Pierson SK, Stonestrom AJ, Shilling D, Ruth J, Nabel CS, Singh A (2018). Plasma proteomics identifies a “chemokine storm” in idiopathic multicentric Castleman disease. Am J Hematol.

[4] Liu AY, Nabel CS, Finkelman BS, Ruth JR, Kurzrock R, van Rhee F (2016). Idiopathic multicentric Castleman’s disease: a systematic literature review. Lancet Haematol.

[5] Dispenzieri A, Armitage JO, Loe MJ, Geyer SM, Allred J, Camoriano JK (2012). The clinical spectrum of Castleman’s disease. Am J Hematol.

[6] Kulaylat AS, Schaefer EW, Messaris E, Hollenbeak CS (2019). Truven Health Analytics MarketScan Databases for Clinical Research in Colon and Rectal Surgery. Clin Colon Rectal Surg.

[7] Mukherjee S, Martin R, Sande B, Paige J, Fajgenbaum DC. Epidemiology and treatment patterns of idiopathic multicentric Castleman disease in the era of IL-6 directed therapy. Blood Adv. 2021:bloodadvances.2021004441. 10.1182/bloodadvances.2021004441. 10.1182/bloodadvances.2021004441 PMC8791564 34535010

[8] Fajgenbaum DC, Uldrick TS, Bagg A, Frank D, Wu D, Srkalovic G (2017). International, evidence-based consensus diagnostic criteria for HHV-8-negative/idiopathic multicentric Castleman disease. Blood.

[9] Simpson D (2018). Epidemiology of Castleman disease. Hematol Clin.

[10] Pierson SK, Khor JS, Ziglar J, Liu A, Floess K, NaPier E (2020). ACCELERATE: a Patient-Powered Natural History Study Design Enabling Clinical and Therapeutic Discoveries in a Rare Disorder. Cell Rep Med.

