# Correction to: Organ dysfunction, thrombotic events, and malignancies in patients with idiopathic multicentric Castleman disease: a population-level US health claims analysis

**DOI:** 10.1038/s41375-022-01791-y

**Published:** 2022-12-16

**Authors:** Sudipto Mukherjee, Karan Kanhai, David Kauffman, Rabecka Martin, Jeremy S. Paige, Anirvan Ghosh, Hannah Kannan, Francis Shupo, David C. Fajgenbaum

**Affiliations:** 1 Department of Hematology and Medical Oncology, Taussig Cancer Institute, Cleveland Clinic, Cleveland, OH USA; 2 EUSA Pharma, Hemel Hempstead, UK; 3 Eversana, LLC, Milwaukee, WI USA; 4 EUSA Pharma, Burlington, MA USA; 5 Department of Medicine, Perelman School of Medicine, University of Pennsylvania, Philadelphia, PA USA

**Keywords:** Haematological diseases, Lymphoproliferative disorders

Correction to: *Leukemia* 10.1038/s41375-022-01690-2, published online 29 September 2022

The article “Organ dysfunction, thrombotic events, and malignancies in patients with idiopathic multicentric Castleman disease: a population-level US health claims analysis”, written by Sudipto Mukherjee, Karan Kanhai, David Kauffman, Rabecka Martin, Jeremy S. Paige, Anirvan Ghosh, Hannah Kannan, Francis Shupo and David C. Fajgenbaum, was originally published Online First without Open Access. After publication in volume 36, issue 10, pages 2539–2543 the author decided to opt for Open Choice and to make the article an Open Access publication. Therefore, the copyright of the article has been changed to © The Author(s) 2022 and the article is forthwith distributed under the terms of the Creative Commons Attribution 4.0 International License, which permits use, sharing, adaptation, distribution, and reproduction in any medium or format, as long as you give appropriate credit to the original author(s) and the source, provide a link to the Creative Commons licence, and indicate if changes were made. The images or other third-party material in this article are included in the article’s Creative Commons licence, unless indicated otherwise in a credit line to the material. If material is not included in the article’s Creative Commons licence and your intended use is not permitted by statutory regulation or exceeds the permitted use, you will need to obtain permission directly from the copyright holder. To view a copy of this licence, visit http://creativecommons.org/licenses/by/4.0/.

